# Sulfur-Induced Thiol Metabolism Confers Arsenic Stress Tolerance Despite Increased Arsenic Accumulation in Rice Seedlings

**DOI:** 10.3390/antiox15101331

**Published:** 2026-10-15

**Authors:** Ha-il Jung, Myung-Sook Kim, Eun-Jin Lee, Tae-Gu Lee, Theint Thandar Latt, Jinwook Lee, Chaw Su Lwin, Bok-Rye Lee

**Affiliations:** 1 Soil and Water Environment Division, National Institute of Agricultural Sciences, Rural Development Administration, Wanju 55365, Republic of Korea; hj255@korea.kr (H.-i.J.); msk74@korea.kr (M.-S.K.); eunjin0219@korea.kr (E.-J.L.); leetg7942@korea.kr (T.-G.L.); 2 Department of Plant Science and Technology, Chung-Ang University, Anseong 17546, Republic of Korea; theintthandarlatt@gmail.com (T.T.L.); jl425@cau.ac.kr (J.L.); 3 Institute of Environmentally-Friendly Agriculture (IEFA), Chonnam National University, Gwangju 61186, Republic of Korea

**Keywords:** arsenic, rice, sulfur, detoxification, thiol metabolism

## Abstract

Arsenic (As) contamination poses a serious threat to rice production and food safety. Although sulfur (S) nutrition is known to alleviate As toxicity through thiol-based detoxification, the mechanisms underlying the relationship between S-induced As accumulation and tolerance remain poorly understood. We investigated the effects of contrasting S levels (0, 1, and 4 mM) on As accumulation, translocation, oxidative stress responses, thiol metabolite pools, and mineral nutrient profiles in hydroponically grown rice seedlings exposed to 15 µM As for 15 days. S supplementation alleviated As-induced growth inhibition in a dose-dependent manner despite increasing total As accumulation in both roots and shoots, demonstrating that S-mediated tolerance is independent of reduced As accumulation. Elevated S supply reduced oxidative stress markers (O_2_^•–^, H_2_O_2_, and MDA), while markedly enlarged the pools of γ-glutamylcysteine (γ-EC), glutathione, and phytochelatins (PCs), particularly in roots. S supplementation also improved the uptake of major essential nutrients, including nitrogen and potassium in shoots, while selectively modulating micronutrient distributions under As stress. Correlation analysis showed that root As content was strongly and positively correlated with γ-EC and PC2–PC4, and principal component analysis separated the treatments along the S gradient in both roots and shoots. Collectively, these results suggest that S enhances As tolerance in rice seedlings through thiol-dependent detoxification, thereby decoupling As tolerance from As accumulation rather than by restricting As accumulation.

## 1. Introduction

Arsenic (As) is a toxic metalloid widely dispersed in soil and water systems [1,2], and it is recognized as a major global contaminant with well-documented carcinogenic risks to human health [3]. Rice (*Oryza sativa* L.) is particularly vulnerable to As contamination because it accumulates As more efficiently than most other cereal crops, primarily due to the anaerobic conditions of flooded paddy soils [4]. Under such conditions, a reductive dissolution of Fe oxides releases As into the soil solution, and the concurrent reduction of arsenate [As(V)] to arsenite [As(III)] increases As bioavailability in the rhizosphere [5,6,7]. The high efficiency of Si-transporters (Lsi1 and Lsi2) in rice roots facilitates the rapid entry of As(III) into plant tissues [8], where it triggers the overproduction of reactive oxygen species (ROS), including superoxide anion radicals (O_2_^•–^), hydrogen peroxide (H_2_O_2_), and hydroxyl radicals (•OH), leading to severe oxidative damage and membrane dysfunction [9]. Furthermore, As exposure impairs mineral homeostasis by competing with essential nutrients and compromising root integrity, thereby suppressing the acquisition of critical macro- and micronutrients such as potassium (K), nitrogen (N), zinc (Zn), and manganese (Mn) [10]. Recent evidence indicates that plant tolerance to As is associated with coordinated regulation of antioxidant defense, iron and arsenic uptake, nitrogen metabolism, and sulfur assimilation, highlighting the interconnected nature of redox and nutrient responses to As stress [11]. Given that the mitigation of oxidative stress and nutrient imbalances largely depends on sulfur-containing ligands and antioxidant enzymes, sulfur (S) nutrition emerges as a decisive factor in modulating As phytotoxicity and enhancing plant resilience.

S is a fundamental macronutrient that underpins the primary defense system against As toxicity in plants. Following its uptake and assimilation, S is incorporated into cysteine, the essential precursor for the biosynthesis of thiol-rich compounds, including glutathione (GSH) and phytochelatins (PCs) [12]. GSH functions as a pivotal cellular redox buffer, directly scavenging As-induced ROS, while simultaneously serving as the substrate for PC synthesis [13]. These PCs have been reported to bind As to form stable As-thiol complexes, which are then sequestered into the vacuole [14,15,16]. Recent evidence has also demonstrated that nano-enabled treatments can alleviate As toxicity by enhancing antioxidant defense and phytochelatin-associated detoxification, highlighting the importance of redox regulation and thiol metabolism in plant responses to As stress [17]. Beyond sequestration, GSH-mediated redox regulation is critical for maintaining the structural and functional integrity of the plasma membrane, which may safeguard mineral nutrient transporters from As-induced oxidative impairment [13]. Consequently, S availability enhances thiol-dependent As detoxification and maintains cellular homeostasis, collectively improving plant tolerance to As toxicity [18].

While these thiol-based mechanisms are well established in alleviating As toxicity, their influence on As mobility and accumulation remain inconsistent across studies. Several studies have demonstrated that S application effectively restricts As bioavailability and uptake by facilitating the formation of stable As_2_S_3_ and FeAsS precipitates [19,20,21]. Conversely, others have reported tissue-specific responses, whereby S fertilization reduces As translocation to the shoots while paradoxically increasing root As accumulation [22,23]. Most significantly, recent evidence indicates that S can enhance As stress tolerance despite promoting higher As accumulation [24,25], suggesting that S-mediated detoxification may operate through internal physiological mechanisms independent of As exclusion. However, the thiol-mediated pathways and antioxidant responses underlying this decoupling of accumulation and tolerance remain poorly understood. Furthermore, the integrative physiological responses linking S nutrition, thiol metabolism, and oxidative stress under As exposure have not been systematically characterized in rice.

We have therefore hypothesized that S supplementation enhances thiol-dependent detoxification and antioxidant buffering capacity in rice seedlings under As stress, thereby mitigating oxidative damage and preserving physiological functions and mineral nutrient distribution in a tissue- and element-specific manner, that is, sustaining As tolerance irrespective of changes in As accumulation. To test this hypothesis, hydroponically grown rice seedlings supplied with contrasting S levels were exposed to As, and their growth responses, As concentration and accumulation, oxidative stress markers, thiol metabolite pools (γ-glutamylcysteine [γ-EC], GSH, and PCs), and mineral nutrient profiles were evaluated. Furthermore, multivariate analyses, including principal component analysis (PCA) and correlation network construction, were also employed to elucidate the integrative relationships among these response variables.

## 2. Materials and Methods

### 2.1. Hydroponic Culture Conditions and Experimental Setup

Rice (*Oryza sativa* L. ssp. *japonica* cv. Shindongjin) seeds were obtained from the National Institute of Crop and Food Science, Rural Development Administration (Wanju, Republic of Korea). Seeds were disinfected in 70% ethanol for 2 min and rinsed five times with distilled water. They were subsequently soaked in 8% ipconazole solution for 48 h, washed thoroughly, and germinated in deionized water at 30 °C for 48 h. Uniform seedlings were selected and transferred to a hydroponic culture system until the two-leaf stage. Eight seedlings were placed in each plastic container (30 × 25 × 15 cm; 0.075 m^2^) containing 9 L of nutrient solution. The standard nutrient solution contained 1 mM S, 1.5 mM NH_4_NO_3_, 0.505 mM KH_2_PO_4_, 0.045 mM K_2_HPO_4_, 4 mM KCl, 3 mM CaCl_2_, 1 mM MgCl_2_, 1 mM MgSO_4_, 0.4 mM Fe-EDTA, 14 μM H_3_BO_3_, 5 μM MnCl_2_, 0.7 μM CuCl_2_, 3 μM ZnCl_2_, and 0.7 μM (NH_4_)_6_Mo_7_O_24_ [13,26]. The S treatments were prepared by replacing sulfate salts with chloride salts while maintaining constant Mg^2+^ and K^+^ concentrations among treatments. For the 0 mM S treatment, MgSO_4_ was omitted and MgCl_2_ was adjusted to maintain the Mg^2+^ concentration. For the 4 mM S treatment, KCl and MgCl_2_ were replaced with K_2_SO_4_ and MgSO_4_, respectively. Although Cl^−^ concentrations varied among S treatments, K^+^ and Mg^2+^ concentrations were maintained at identical levels across all treatments. The resulting concentrations of the major ions in each treatment are presented in Table S1. Plants were cultivated in a greenhouse at the National Institute of Agricultural Sciences (Rural Development Administration, Wanju, Republic of Korea) under natural daylight. The temperature was maintained at 30 °C during the day and 25 °C at night, with relative humidity of 60% and 80%, respectively. Three biological replicates (containers) were prepared for each treatment, and container positions were changed daily. When the plants reached the five-leaf stage, As stress was imposed by adding 15 μM As(III) (NaAsO_2_) to the nutrient solution together with 0, 1, or 4 mM S for 15 days. Nutrient solution in each pot was completely replaced every 5 days. At each replacement, freshly prepared nutrient solutions containing the designated S concentrations (0, 1, or 4 mM) and 15 μM As(III) (NaAsO_2_) were supplied to the corresponding pots. At harvest, roots and leaves were collected, immediately frozen in liquid nitrogen, and stored at −80 °C until biochemical analyses. For destructive measurements, seedlings within each pot were harvested and pooled as a composite sample per pot.

### 2.2. Rice Seedling Growth Under as Stress

Plant growth was evaluated using plant height, shoot dry weight, and root dry weight. Following the 15-day treatment period, plants were removed from the nutrient solution and separated into shoots and roots. Plant height was determined from the stem base to the tip of the longest leaf. The separated tissues were dried at 80 °C for 72 h until their weights remained constant, after which dry weight was recorded.

### 2.3. Quantification of Total As and Mineral Nutrient Contents

Harvested roots and shoots were washed with running tap water and subsequently rinsed five times with distilled water to remove externally attached materials. For elemental analysis, 200 mg of powdered tissue was subjected to acid digestion using a graphite-block digestion system (ODLAB Co., Ltd., Seoul, Republic of Korea). After digestion, the samples were cooled, brought to a final volume of 100 mL with ultrapure water, and passed through Whatman Grade 40 filter paper (Whatman International Ltd., Buckinghamshire, UK). Arsenic and mineral elements, including P, K, Ca, Mg, Si, Fe, Mn, Cu, Zn, B, and Mo, were quantified by inductively coupled plasma–mass spectrometry (ICP-MS; Agilent 7900, Agilent Technologies Inc., Santa Clara, CA, USA). Individual As species were not determined. Total N and S concentrations were quantified separately using a CNS elemental analyzer (Vario MAX CNS, Elementar Analysensysteme GmbH, Langenselbold, Germany). As concentration in root and shoot tissues is expressed on a dry weight basis. Total As accumulation was calculated according to the following equation:

*As accumulation* = *Ca* × *Cb*

where *Ca* represents the As concentration (mg kg^−1^ DW) and *Cb* is the corresponding dry biomass (g plant^−1^) of the root or shoot.

### 2.4. Quantification of ROS Production and Lipid Peroxidation

Superoxide anion radical (O_2_•^−^) was determined using the hydroxylamine oxidation procedure of Elstner and Heupel [27]. Hydroxylamine hydrochloride was added to the extracts, and the resulting nitrite was quantified spectrophotometrically at 530 nm. Superoxide anion radical concentrations were obtained from a NaNO_2_ calibration curve. Hydrogen peroxide (H_2_O_2_) was quantified using the colorimetric procedure described previously [28]. Absorbance of the reaction mixture was measured at 410 nm, and H_2_O_2_ concentration was calculated using an extinction coefficient of 0.28 μM^−1^ cm^−1^. Lipid peroxidation was estimated from thiobarbituric acid-reactive substances (TBARS) following a modified protocol [29]. The reaction mixtures were measured at 532 nm, while the absorbance at 600 nm was used to correct for nonspecific turbidity. MDA concentration was calculated using an extinction coefficient of 156 mM^−1^ cm^−1^.

### 2.5. Quantitative Determination of γ-Glutamylcysteine (γ-EC), Glutathione (GSH), and Phytochelatins (PC2, PC3, and PC4)

Approximately 250 mg of fresh root or shoot tissue was homogenized with 300 μL of chilled extraction solution containing 5% (*w*/*v*) 5-sulfosalicylic acid, 6.3 mM diethylenetriaminepentaacetic acid, and 2 mM tris(2-carboxyethyl)phosphine [30,31]. The homogenates were vortex-mixed for 30 s and mechanically disrupted using a Mixer Mill MM 400 (Retsch GmbH, Haan, Germany) for 5 min at 30 s^−1^. Samples were then kept on ice for 15 min. A further 5 min milling step was performed under the same conditions. The extracts were centrifuged at 13,000 rpm for 10 min at 4 °C. The resulting supernatants were passed through 0.22 μm membrane filters. γ-EC, GSH, and phytochelatins (PC2, PC3, and PC4) were quantified by liquid chromatography–tandem mass spectrometry using a Nexera X2 UHPLC system (Shimadzu Corp., Kyoto, Japan) coupled with a triple-quadrupole mass spectrometer. Instrumental conditions, including collision energies and multiple-reaction-monitoring transitions, were optimized for rice tissue extracts according to a previously established method [32].

### 2.6. Multivariate Statistical Analysis and Network Visualization

Relationships among growth traits, oxidative stress indicators, thiol metabolites, As accumulation, and mineral elements were examined using multivariate statistical approaches. The dataset included measurements obtained from roots and shoots of plants exposed to the three S concentrations (0, 1, and 4 mM), both in the presence and absence of As. Before analysis, the variables were autoscaled. Pearson correlation coefficients were calculated using MetaboAnalyst v6.0 and visualized as correlation matrices and normalized heatmaps [33]. Principal component analysis (PCA) was subsequently performed to evaluate sample distribution and identify variables associated with treatment-related separation. Correlation networks were constructed with Cytoscape v3.8.2 using the MetScape v3.1.3 plugin [34]. In these networks, nodes represented the measured parameters, whereas edges represented statistically significant correlations, providing an integrated visualization of the interactions among growth, oxidative stress, thiol metabolism, As accumulation, and mineral nutrient distribution in response to S supplementation under As stress.

### 2.7. Statistical Analysis

All data were analyzed using two-way analysis of variance (ANOVA) with SAS version 9.2 (SAS Institute Inc., Cary, NC, USA). For mean separation, Duncan’s multiple range test (DMRT) was used when treatment effects were significant (*p* < 0.05). All figures display the results as mean ± standard deviation (SD).

## 3. Results

### 3.1. Sulfur Supplementation Alleviated As-Induced Growth Inhibition in Rice Seedlings

**Figure 1:**
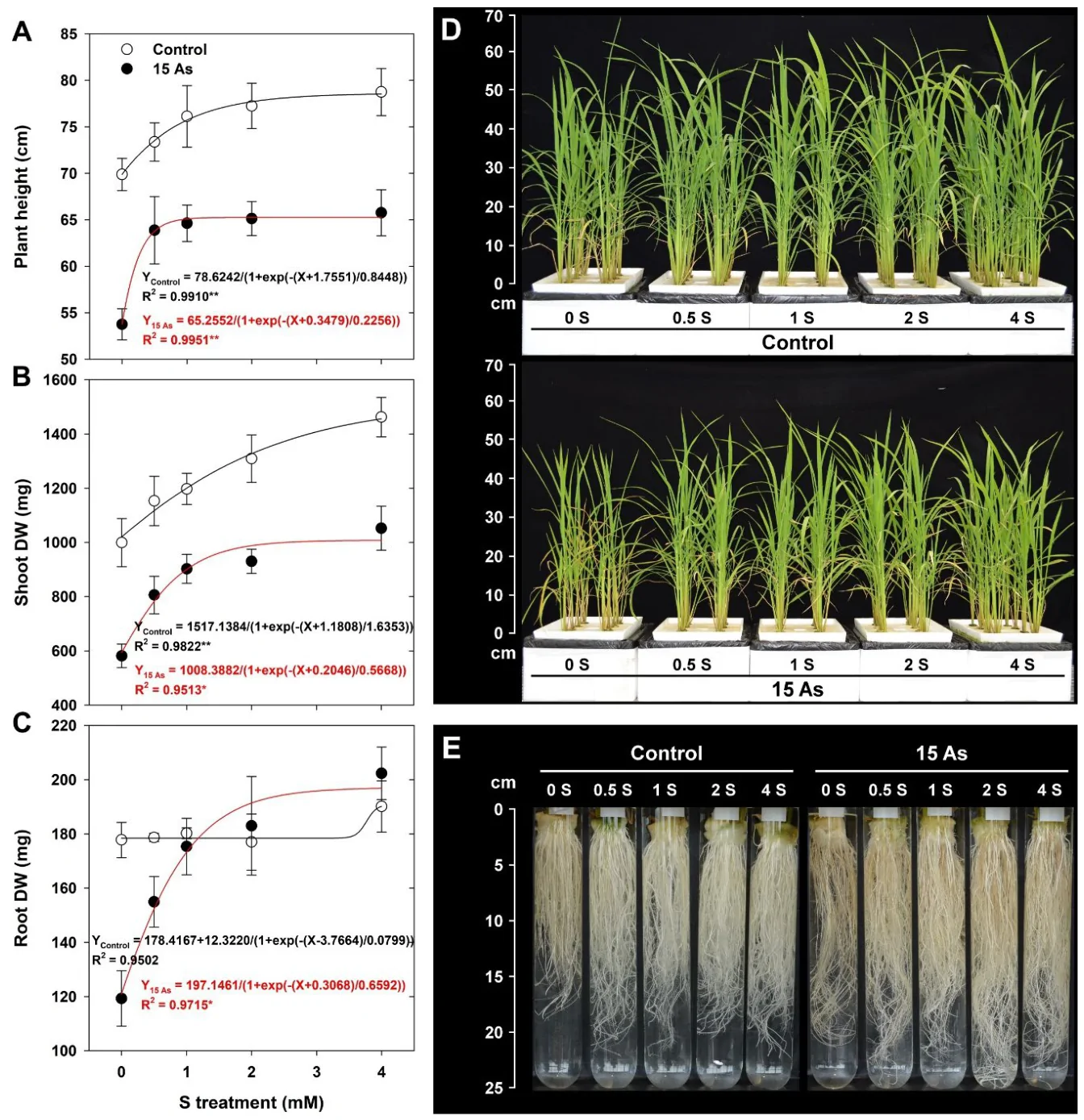
Effect of sulfur (S) supplementation on growth parameters of rice seedlings grown under arsenic (As)-treated hydroponic conditions. (**A**) Plant height, (**B**) shoot dry weight (DW), and (**C**) root DW. Representative images of (**D**) shoots and (**E**) roots at 15 days after treatment. Data are presented as mean ± standard deviation (*n* = 3). Asterisks indicate significance at * *p* < 0.05 and ** *p* <0.01.

Rice seedlings exposed to As exhibited significant reductions in plant height, shoot DW, and root DW, compared with untreated controls (Figure 1A–C). These growth reductions were accompanied by visible symptoms of toxicity, including chlorotic leaf tips, reduced stem elongation, and stunted roots (Figure 1D,E). Under As exposure, plant growth was markedly reduced under S deficiency (0 mM), whereas additional S supply from 1 to 4 mM improved plant growth. Plant height increased with increasing S concentration, while shoot DW, which was markedly reduced under As stress, was progressively restored and approached the control level at 4 mM S. Root DW also increased with S supplementation, indicating enhanced root growth and nutrient uptake under As stress (Figure 1A–C). The most substantial improvements in biomass were observed at 2 mM and 4 mM S, indicating that higher S availability enhances rice tolerance to As. Morphologically, S-treated seedlings regained deep green foliage, produced sturdier stems and internodes, and developed more lateral roots, consistent with the quantitative growth data. Two-way ANOVA showed that As and S each significantly affected growth (Table S2). A significant As × S interaction was found for root DW (*F* = 27.75, *p* < 0.001), which increased with S only under As stress (119 to 202 mg), whereas shoot DW showed no significant interaction (*p* = 0.253). These findings highlight the essential role of S in counteracting As-induced growth suppression, likely through improved nutrient assimilation and detoxification, and support the use of S fertilization as a strategy to sustain rice performance in As-contaminated soils.

### 3.2. Sulfur Supplementation Dose-Dependently Elevated Total As Concentration and Accumulation in Rice Roots and Shoots

**Figure 2:**
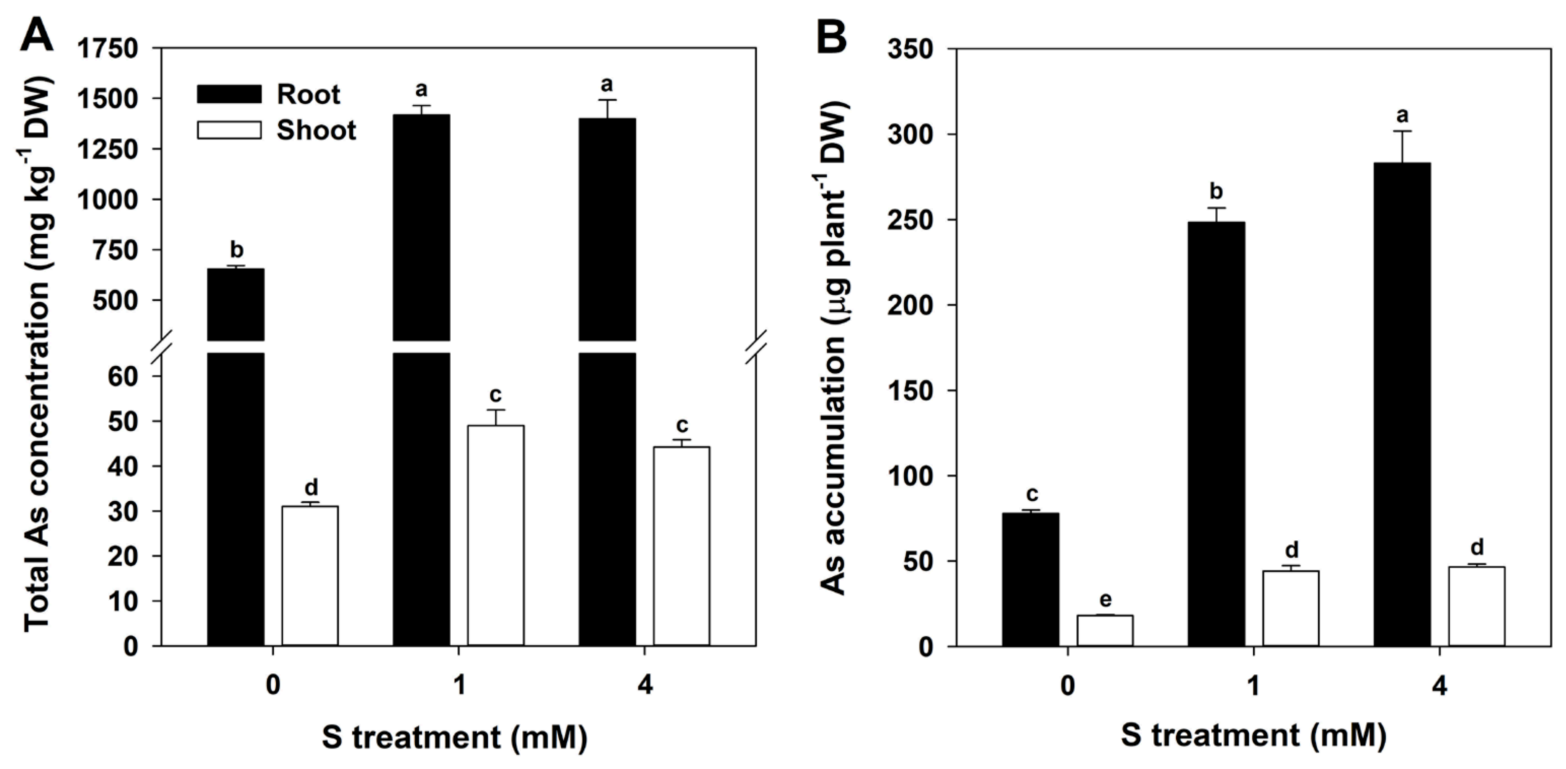
Influence of sulfur (S) supplementation on total arsenic (As) concentration (**A**) and As accumulation (**B**) in rice plants cultured in As-treated hydroponic conditions. Data are presented as mean ± standard deviation (*n* = 3). Values sharing the same letter are not significantly different at *p* < 0.05.

Total As concentration and accumulation in As-treated plants were significantly affected by S in both roots and shoots (one-way ANOVA, *p* < 0.001; Table S2). S supplementation markedly increased total As concentrations in both root and shoot tissues of rice seedlings in a dose-dependent manner (Figure 2A). In roots, total As concentration almost doubled at 1 mM S, compared with that of the untreated control and remained at this elevated level at 4 mM. Shoots displayed a similar trend, with As concentrations rising steadily to a peak at 1 mM S and showing only slight differences between 1 mM and 4 mM S treatments. Total As accumulation per plant closely paralleled the tissue-specific responses, increasing progressively with increasing S supply (Figure 2B). Seedlings treated with 1 mM S accumulated approximately three-fold higher As compared with controls, whereas those receiving 4 mM S exhibited the highest root As accumulation. These results demonstrate that S fertilization enhances total As accumulation in rice. Although increased As accumulation would be undesirable in rice intended for food production, this response may warrant further investigation in non-food rice-based phytomediation systems for severely As-contaminated paddy soils.

### 3.3. Sulfur Supplementation Alleviated As-Triggered Oxidative Stress in Rice Seedlings

**Figure 3:**
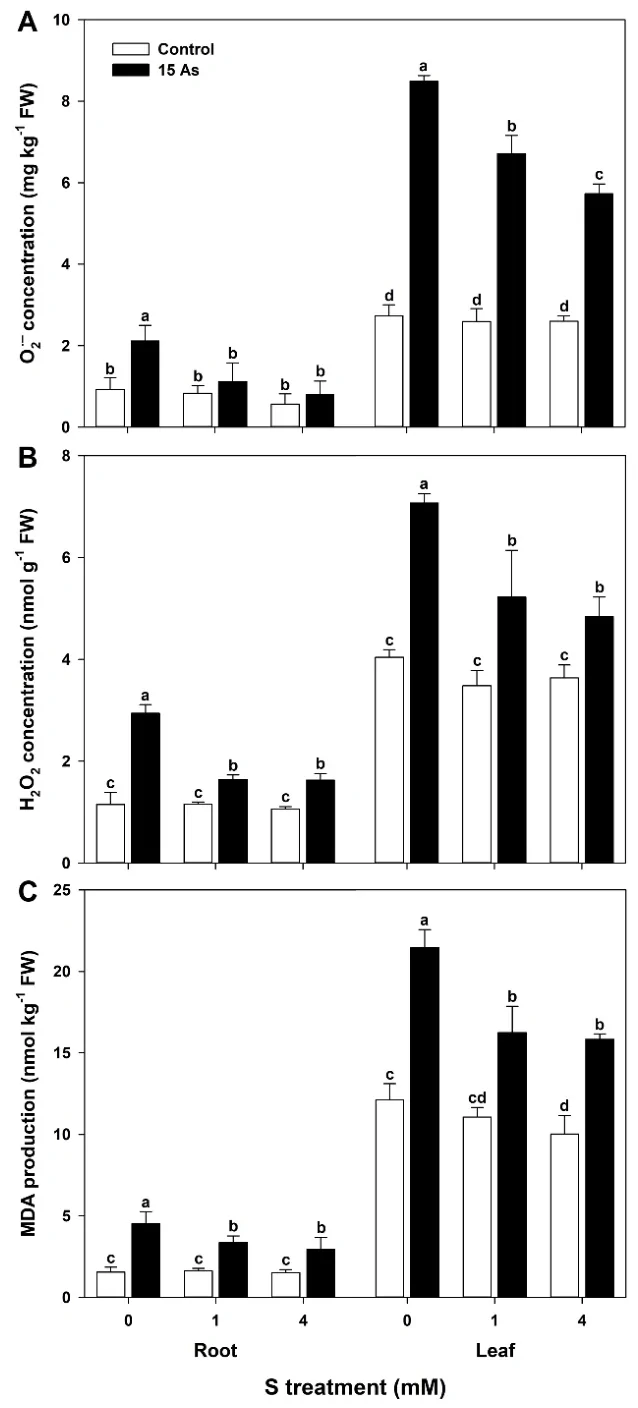
Influence of sulfur (S) supplementation on (**A**) superoxide anion radical (O_2_^•–^), (**B**) hydrogen peroxide (H_2_O_2_), and (**C**) malondialdehyde (MDA) levels in the roots and leaves of rice plants cultivated under control (non-arsenic) and arsenic (As)-treated hydroponic conditions. Data are presented as mean ± standard deviation (*n* = 3). Values sharing the same letter are not significantly different at *p* < 0.05.

Exposure to As significantly increased ROS production, particularly O_2_^•–^ and H_2_O_2_, in both root and leaf tissues, with the highest levels observed in seedlings grown without S supplementation (Figure 3A,B). This oxidative surge coincided with elevated MDA accumulation, indicating enhanced lipid peroxidation and compromised membrane stability (Figure 3C). Supplying S markedly decreased ROS and MDA levels in As-exposed seedlings (Figure 3A–C). Supplemental S (4 mM) further lowered both oxidative markers beyond the adequate baseline (1 mM S), indicating mitigated lipid peroxidation. As and S significantly affected O_2_^•–^, H_2_O_2_, and MDA in both roots and shoots, with significant As × S interactions (*p* < 0.05; Table S2). These results demonstrate that adequate S supplementation was associated with reduced oxidative stress and preserves membrane integrity in rice seedlings exposed to As.

### 3.4. Sulfur-Enhanced Thiol Metabolism and PC Biosynthesis in As-Exposed Rice Seedlings

**Figure 4:**
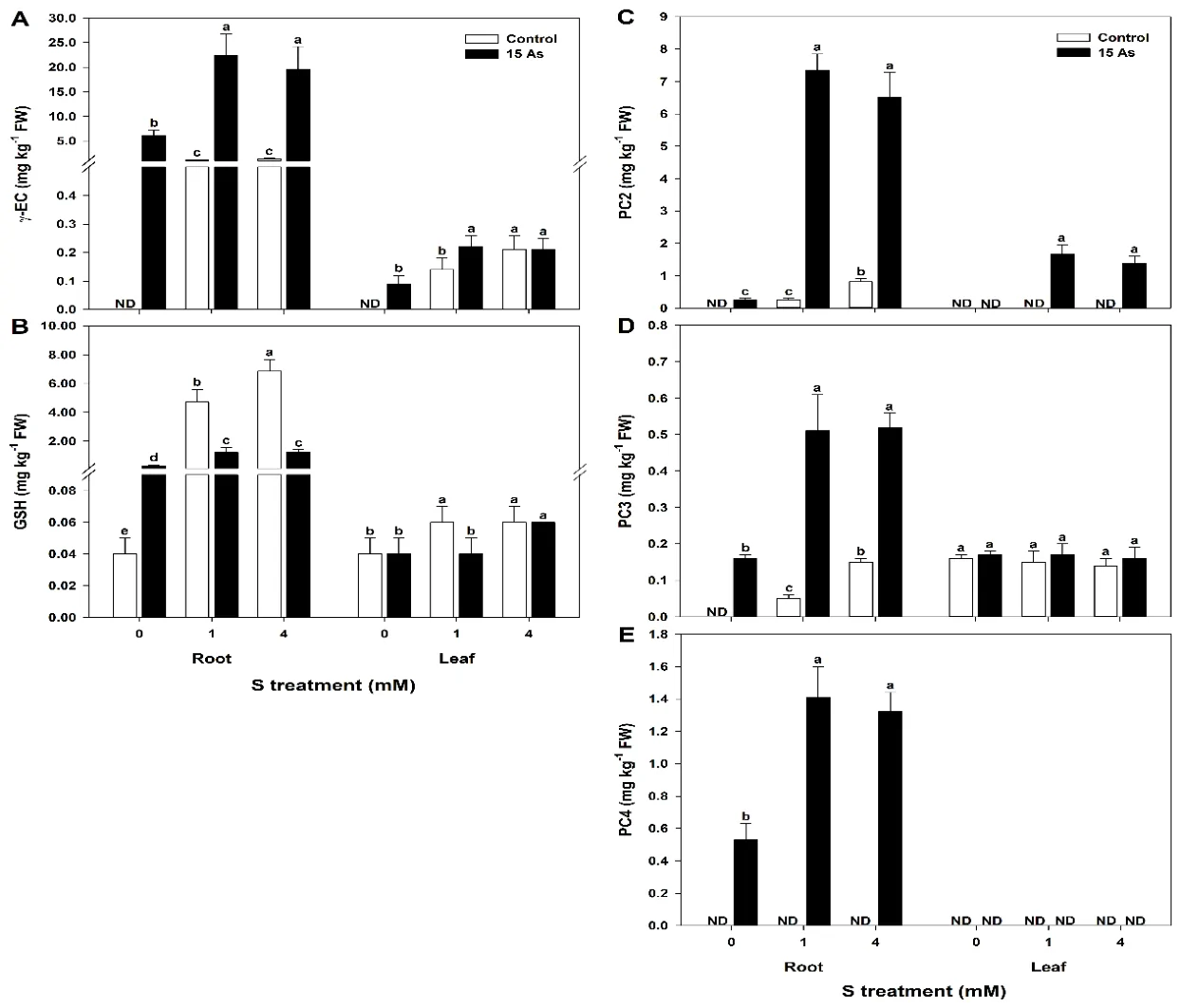
Influence of sulfur (S) supplementation on (**A**) gamma-glutamylcysteine (γ-EC), (**B**) glutathione (GSH), and (**C**–**E**) phytochelatin contents such as (**C**) PC2, (**D**) PC3, and (**E**) PC4 in the roots and leaves of rice plants grown in control and arsenic (As)-treated hydroponic conditions. Data are presented as mean ± standard deviation (*n* = 3). Values sharing the same letter are not significantly different at *p* < 0.05. ND, not detected.

Under As-free conditions, γ-EC levels in both roots and leaves increased with increasing S supply, reaching the highest levels at 1 mM in roots and 4 mM in leaves (Figure 4A). When seedlings were exposed to As, γ-EC concentrations increased further at all S concentrations, compared with those in the corresponding As-free controls (Figure 4A). In contrast, GSH accumulation was increased with increasing S supply under As-free conditions, reaching the highest level at 4 mM S. However, GSH concentrations declined markedly under As stress across all S treatments (Figure 4B). These results indicate that As exposure differentially affected γ-EC and GSH accumulation, with γ-EC increasing and GSH decreasing relative to the corresponding As-free conditions.

Furthermore, PCs are cysteine-rich, low-molecular-weight polypeptides synthesized by PC synthase using GSH as a substrate, with the formula (γ-Glu-Cys)_n_-Gly, where n can vary from 2 to 11 [35]. PCs play an important role in detoxifying metals in plants [36]. In As-free seedlings, PC2, PC3, and PC4 were undetectable in both roots and leaves. Increasing S concentration led to stepwise increases in PC2 and PC3 (Figure 4C–E). Under As stress, all three PCs were highly upregulated in roots, with PC2 showing the strongest S-dependent induction (Figure 4C–E). PC2 levels in S-treated leaves were markedly higher than those in untreated controls but did not differ significantly between 1 mM and 4 mM S treatments (Figure 4C). PC3 remained unchanged across treatments, whereas PC4 was undetectable under all conditions (Figure 4D,E). The As × S interaction was significant for γ-EC, GSH, PC2, PC3, and PC4 in roots and for PC2 in shoots (*p* < 0.001), but not for γ-EC (*p* = 0.078), GSH (*p* = 0.105), or PC3 (*p* = 0.932) in shoots (Table S2). These results indicate that PC2 is the predominant phytochelatin isoform induced by S supplementation under As exposure in both roots and leaves. This pronounced accumulation suggests that PC2 may contribute to thiol-mediated As detoxification, although intact As–PC2 complex formation and its specific subcellular localization remain to be directly quantified.

### 3.5. Sulfur Supplementation Altered Macro- and Micronutrient Profiles in Rice Seedlings Under As Stress

**Figure 5:**
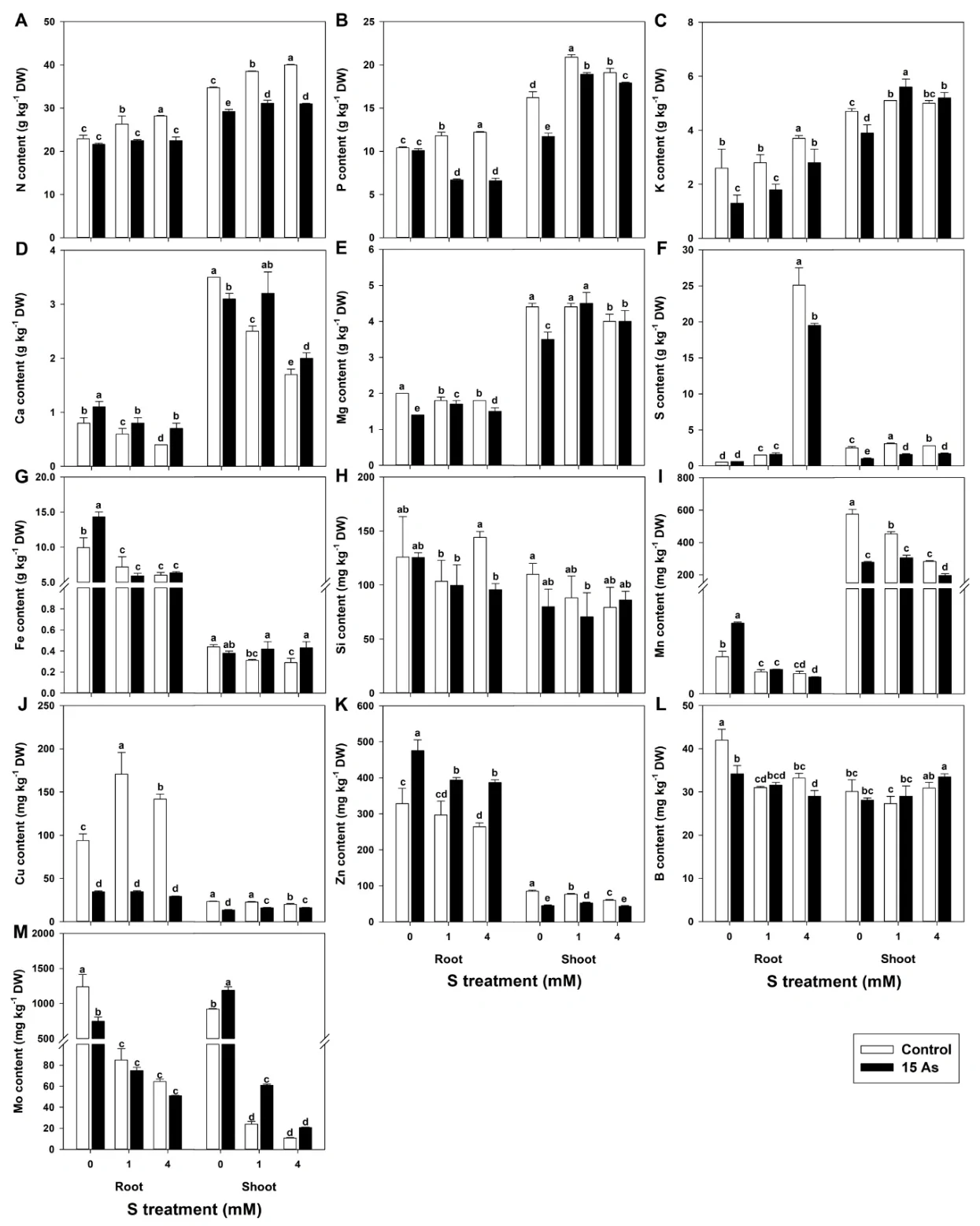
Influence of sulfur (S) supplementation on macro- and micro-nutrient contents in roots and shoots of rice seedlings grown under control and arsenic (As)-treated hydroponic culture. (**A**) Nitrogen (N), (**B**) Phosphorus (P), (**C**) Potassium (K), (**D**) Calcium (Ca), (**E**) Magnesium (Mg), (**F**) Sulfur (S), (**G**) Iron (Fe), (**H**) Silicon (Si), (**I**) Manganese (Mn), (**J**) Copper (Cu), (**K**) Zinc (Zn), (**L**) Boron (B), and (**M**) Molybdenum (Mo). Data are presented as mean ± standard deviation (*n* = 3). Values sharing the same letter are not significantly different at *p* < 0.05.

S supplementation markedly altered the mineral nutrient composition of rice seedlings grown hydroponically for 15 days under both control and As stress conditions (Figure 5A–M). Under control conditions, increasing S from 0 to 1 and 4 mM progressively raised N, P, and K concentrations in roots and shoots, with shoot N and P reaching the highest levels among all macronutrients, and shoot K levels surpassing those of seedlings without S supplementation. In contrast, Ca and Mg levels decreased with increasing S supply, whereas tissue S content peaked in roots at 4 mM (Figure 5A–F). Among micronutrients, Fe, Si, Mn, Zn, B, and Mo levels decreased significantly with elevated S, whereas root Cu levels increased. Mo exhibited the opposite trend to S, with the highest concentrations observed in seedlings grown without S supplementation and a marked decline at higher S levels (Figure 5G–M).

Under As stress, tissue S again increased proportionally with S supply, whereas the responses of other mineral nutrients differed from those observed under control conditions. Root N remained unchanged, whereas shoot N increased slightly; root P declined despite a rise in shoot P; K accumulated consistently in both roots and shoots. Ca decreased in both roots and shoots; whereas Mg, instead of declining, increased markedly with higher S (Figure 5A–F). Micronutrient responses under As stress largely paralleled those under control conditions: root Fe declined and shoot Fe remained stable; Si, Mn, Zn, and B decreased; root Cu was maintained and shoot Cu increased; and Mo, initially highest at 0 mM S, dropped sharply with supplemental S (Figure 5G–M). The As × S interaction was significant for most mineral nutrients (*p* < 0.05), except for K, Ca, Si, and Zn in root and Si and B in shoot (Table S2). These findings indicate that S supply altered mineral nutrient profiles in a tissue- and element specific manner, with increases in selected macronutrients and decreases in several micronutrients under As stress.

### 3.6. Insights into S-Mediated Responses to As Stress via Correlation-Based Heatmap Clustering

**Figure 6:**
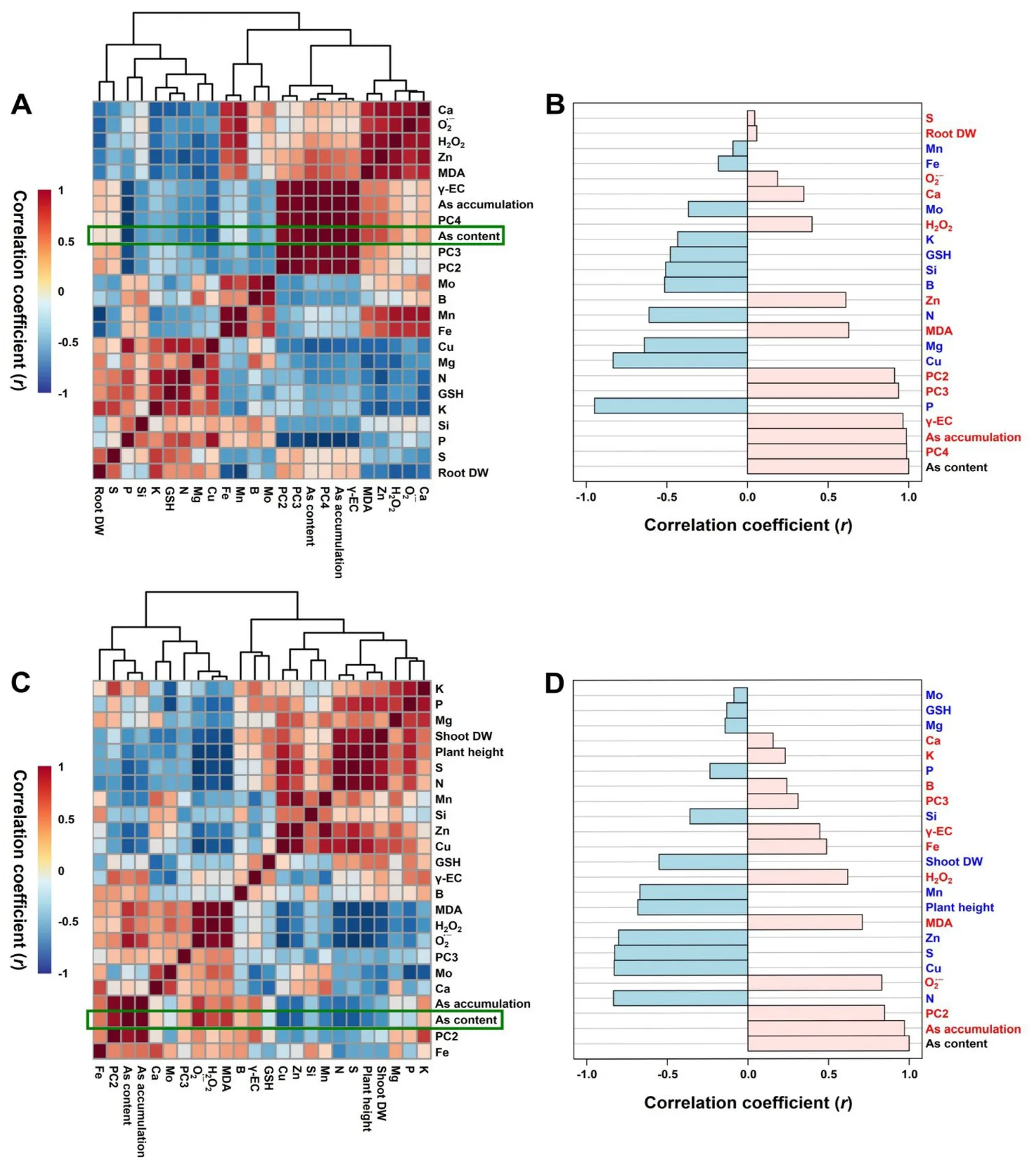
Heatmap responses of Pearson’s correlation coefficients (*r*) among measured parameters, including growth characteristics, oxidative stress, thiol-based detoxification, and mineral nutrient distribution in roots (**A**,**B**) and shoots (**C**,**D**) of sulfur-supplemented rice seedlings subjected to control and arsenic (As) stress. Red denotes positive correlations, whereas blue indicates negative correlations. (**B**,**D**) highlight the correlation coefficients of variables correlated with As concentration in roots and shoots, respectively.

The correlation heatmap of physiological and biochemical responses of S-supplemented rice seedlings under As stress revealed intricate associations among growth parameters, oxidative stress markers (O_2_^•–^, H_2_O_2_, and MDA), thiol-mediated detoxification mechanisms, and the ionic balance of essential mineral nutrients (Figure 6). Correlation-based heatmap clustering of root and shoot datasets identified four distinct groups that, despite minor organ-specific variations, showed consistent correlation patterns and strong positive correlations within clusters (Figure 6A,C). Biomass accumulation correlated positively with macronutrients, such as N, P, K, and S. In contrast, As content showed strong positive associations with ROS-related indicators (O_2_^•–^, H_2_O_2_, and MDA) and low-molecular-weight thiols (γ-EC, GSH, and PCs). Additional clusters showed coherence, particularly among transition metals, such as Cu, Zn, and Mn, which serve as key cofactors for antioxidant enzymes and contribute to cellular redox regulation. In roots, As accumulation was positively associated with PC2–PC4, the GSH precursor (γ-EC), oxidative stress markers (O_2_^•–^, H_2_O_2_, and MDA), and elements, such as Zn and Ca, whereas P, Cu, Mg, N, B, Si, K, Mo, and reduced GSH showed negative correlations (Figure 6B). Similar patterns were observed in leaf tissues (Figure 6D), supporting the view that exogenous S supplementation is associated with coordinated physiological adjustments under As stress, including the co-variation of thiol metabolites, oxidative markers, and nutrient patterns.

### 3.7. Insights into S-Mediated Responses to As Stress via Hierarchical Heatmap Clustering

**Figure 7:**
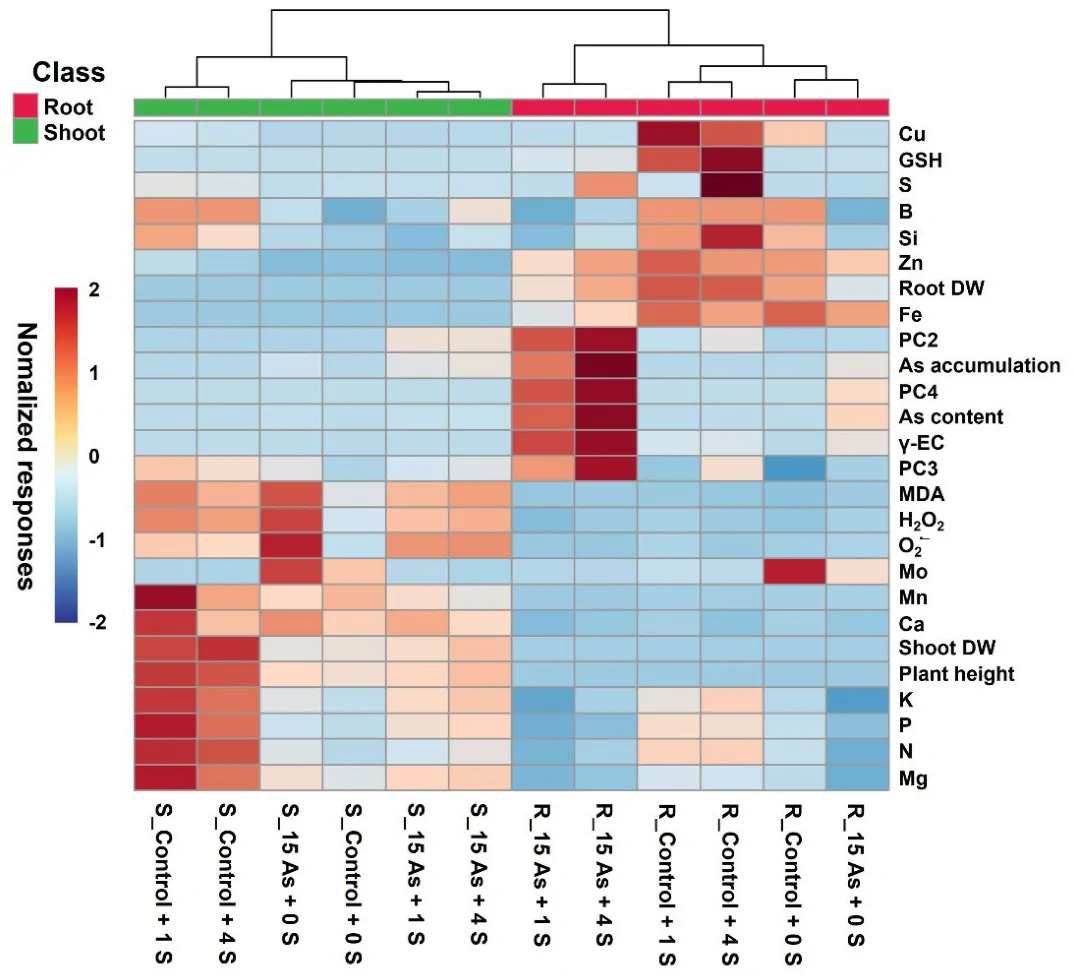
Normalized heatmap matrix showing the responses of rice growth traits, oxidative stress indicators, thiol-based detoxification pathways, and mineral nutrient distribution in roots (R) and shoots (S) of arsenic (As)-stressed plants exposed to different sulfur levels.

Hierarchical clustering of the normalized heatmap matrix revealed two distinct organ-specific response modules in rice exposed to As under various levels of S supplementation (Figure 7). Root samples exposed to As without S supplementation exhibited pronounced growth inhibition, nutrient imbalance, and marked depletion of thiol-related metabolites, including γ-EC, GSH, and PC2–PC4. These disruptions underscore the vulnerability of roots to As toxicity when S is insufficient. Progressive S supplementation at 1 mM and 4 mM significantly ameliorated these effects. At 4 mM S, root biomass and thiol metabolite levels were restored to near-control values, accompanied by enhanced micronutrient uptake. Shoot samples formed a separate cluster, characterized by elevated oxidative stress markers, such as O_2_^•–^, H_2_O_2_, and MDA, and depleted thiol pools under As stress without S. S supplementation progressively reduced oxidative damage, improved shoot growth, and rebalanced nutrient profiles. The clustering pattern revealed an organ-specific distinction, with root showing stronger changes in S-associated thiol metabolism and As tolerance, while shoots benefit from attenuated oxidative stress and adjusted nutrient distribution. These findings provide a strategic basis for optimizing S nutrition to mitigate As toxicity in rice cultivation, where targeted root detoxification may contribute to both stabilizing and removing As from contaminated soils.

### 3.8. Principal Component Analysis of S-Mediated Responses to As Stress

**Figure 8:**
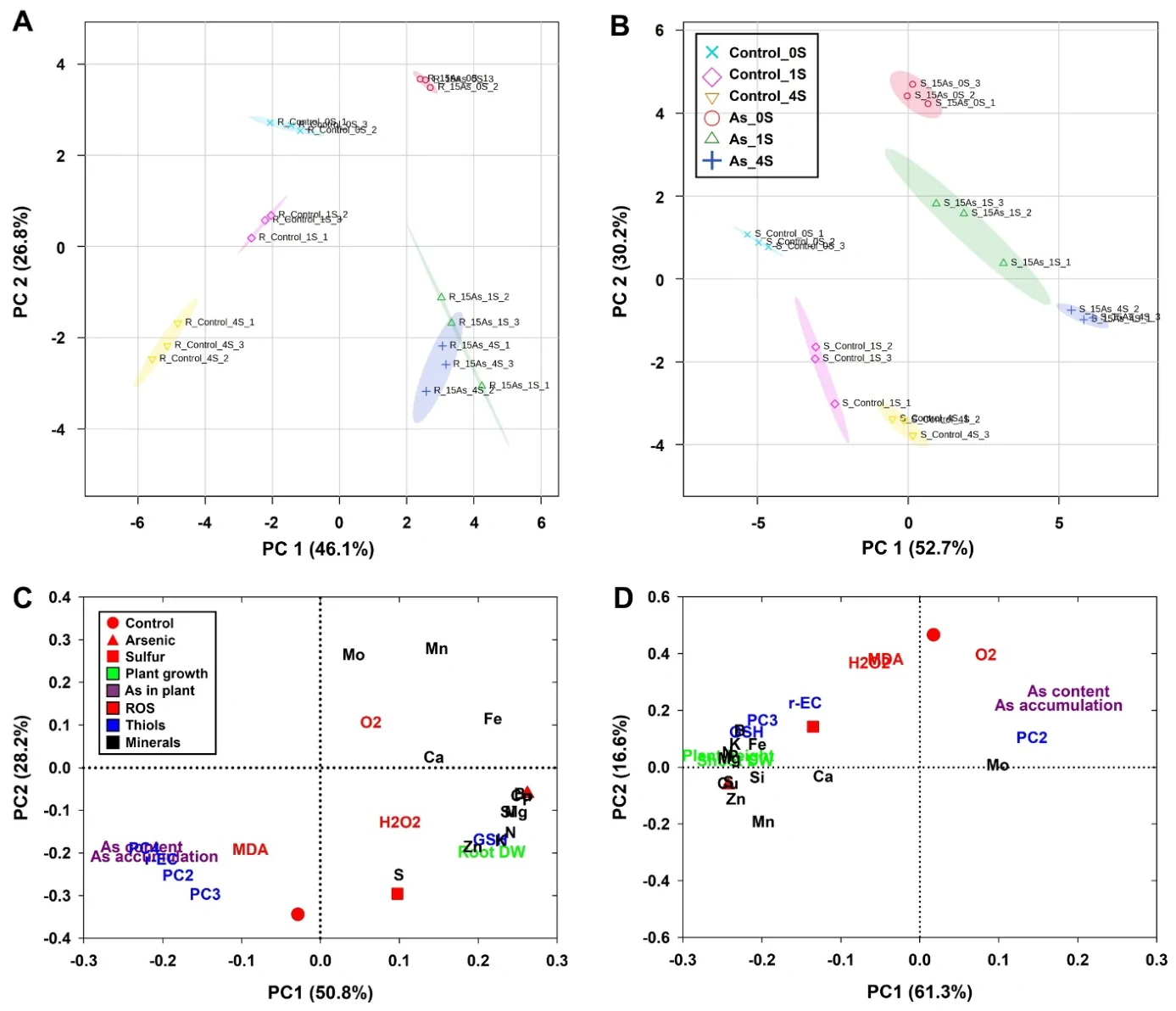
Principal component analysis (PCA) score plots (**A**,**B**) and loading plots (**C**,**D**) for response variables, including growth traits, thiol-based detoxification pathways, mineral nutrient distribution, and oxidative stress in roots (**A**,**C**) and shoots (**B**,**D**) of sulfur-supplemented rice seedlings exposed to control and arsenic (As) stress. Data points represent mean values of three replicates (*n* = 3).

PCA was performed to evaluate the multivariate physiological and biochemical responses of rice seedlings to As stress and S supplementation. In both roots and shoots, PCA score plots distinctly separated the response variables of rice seedlings under As stress from those under non-stress (control) conditions, indicating different physiological and chemical responses (Figure 8). In roots (Figure 8A), PC1 (46.1%) and PC2 (26.8%) separated non-stressed and As-stressed conditions into distinct clusters. Within each group, S supplementation further distinguished the treatments, indicating that both As stress and S supply markedly and differently influenced the response variables. However, under As stress, the 1 mM S and 4 mM S treatments overlapped and were not clearly separated by PCA, suggesting that S supplementation under As stress did not distinctly differentiate variables. In shoots (Figure 8B), PC1 (52.7%) and PC2 (30.2%) provided a stronger separation among groups, indicating a more pronounced differentiation between treatments than in roots.

The loading plots (Figure 8C,D) showed that in roots, As content and accumulation, together with thiol compounds (γ-EC, PC2, PC3, and PC4) and MDA, were associated with negative loadings along PC1. In contrast, H_2_O_2_, root DW, and several nutrients were positioned on the positive side of PC1. In shoots, As-related traits exhibited positive loadings on both PC1 and PC2 axes, whereas growth parameters (plant height and shoot DW), thiol-based compounds, and several nutrients were located opposite to As accumulation along the PC2 axis. This indicates that As-related traits were inversely correlated with growth and nutrient variables. Although As content and accumulation were separated from other variables, their relative associations along PC2 retained the same pattern observed in the root loading plot. Overall, S-treated samples aligned with growth traits, mineral nutrients, and thiol-based compounds, suggesting that S supplementation was associated with distinct physiological and biochemical response patterns under As stress.

### 3.9. Correlation Network Analysis of S-Mediated Responses to As Stress

**Figure 9:**
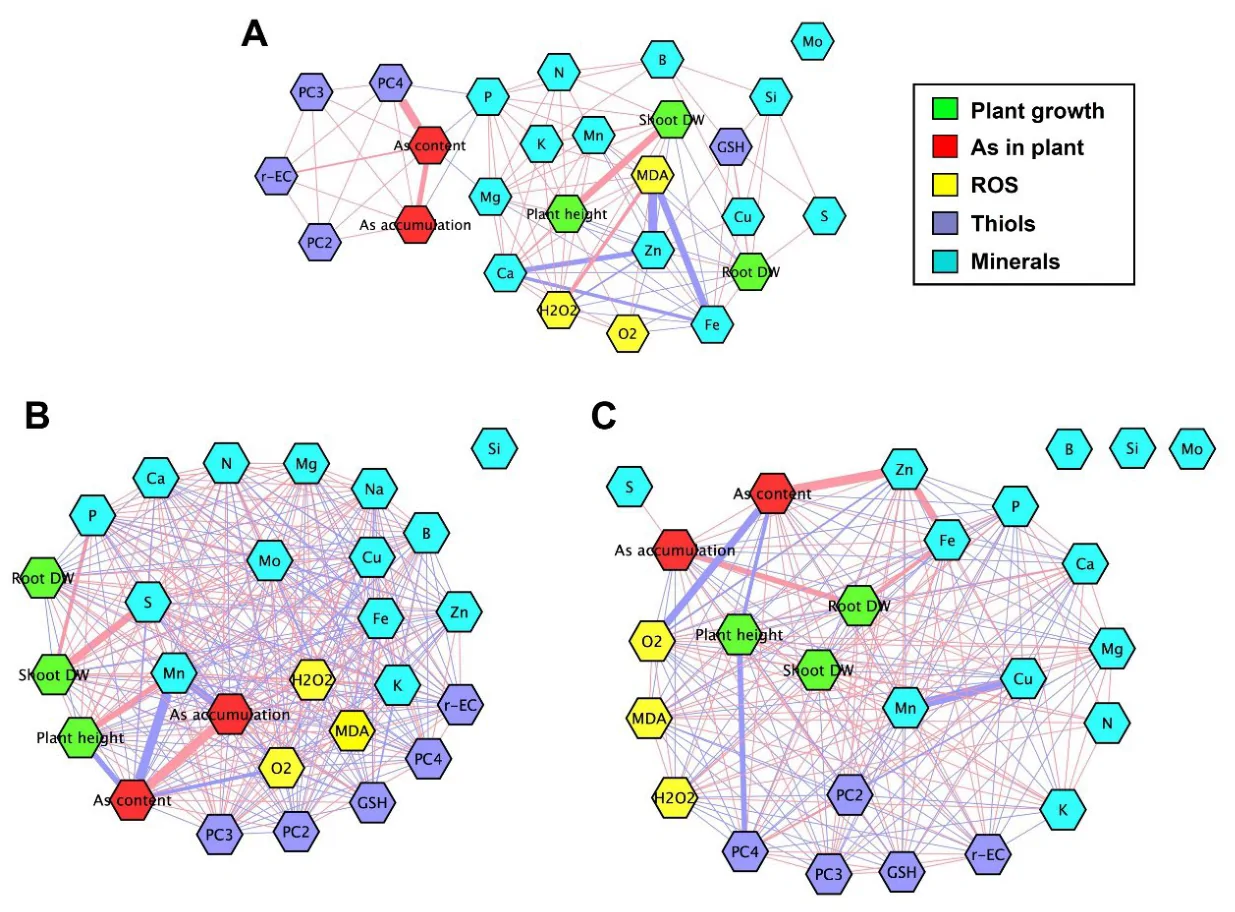
Pairwise correlation coefficient network constructed from the relative changes in response variables in roots and shoots of rice seedlings under three scenarios: (**A**) treated with sulfur (S) under arsenic (As)-stress and non-stress conditions (r ≥ | 0.55 |), (**B**) treated with no S under As-stress conditions (r ≥ | 0.80 |), and (**C**) treated with S under As-stress conditions (r ≥ | 0.80 |). The red and blue colors indicate positive and negative correlations among variables, respectively. The thickness of the correlation lines indicates the strength of the correlation coefficient (r) among variables.

A pairwise correlation coefficient network was constructed to examine the interactions among growth parameters, ROS production, thiol metabolism, As accumulation, and mineral nutrients in rice seedlings (Figure 9). Under both control and As-stress conditions with S supplementation (Figure 9A), two distinct clusters were detected. As accumulation and content were positively grouped with thiol-based compounds, particularly PC4, but they showed negative correlations with P and Mg. In contrast, plant growth traits were strongly associated with most mineral nutrients and oxidative stress markers, forming a separate cluster. MDA was positively correlated with H_2_O_2_ and negatively correlated with Zn, Fe, and Ca. In As-stressed seedlings without S supplementation (Figure 9B), As content and accumulation showed strong negative correlations with Mn, and As content was also negatively correlated with plant height and O_2_^•–^. Shoot DW was positively correlated with S and P. With S supplementation under As stress (Figure 9C), similar patterns were observed: As content showed a negative correlation with plant height and O_2_^•–^ and a positive correlation with Fe and Zn. As accumulation was strongly correlated with root DW and weakly correlated with S, these network patterns highlight the complex associations among S supply, nutrient distribution, thiol metabolism, and oxidative stress in rice seedlings under As exposure.

## 4. Discussion

### 4.1. Sulfur-Enhanced Arsenic Tolerance Is Decoupled from Arsenic Accumulation in Rice Seedlings

Sulfur supplementation significantly alleviated As-induced growth inhibition in rice seedlings (Figure 1). Because 1 mM S represents the basal nutritional requirement, the severe arrest at 0 mM S reflects combined sulfur starvation and As toxicity (Figure 1 and Figure 7). Accordingly, while 1 mM S restored basal growth, supplemental S (4 mM) conferred specific As tolerance beyond nutritional adequacy relative to the 1 mM S under As stress (Figure 1). Notably, S also promoted growth under non-stressed (control) conditions, highlighting its role in enhancing overall plant vigor. Remarkably, this physiological recovery occurred despite significantly elevated total As concentrations in both roots and shoots of S-supplemented seedlings compared with those under As-only treatments (Figure 2A,B). Rather than restricting As accumulation, S supplementation enhanced total As accumulation, particularly within the roots (Figure 2), which aligns with recent reports that S can enhance the retention and potential sequestration of toxic metalloids [20,24,25]. Crucially, elevated total As accumulation in tissues does not necessarily imply an increase in metabolically active, unbound As. If a significant fraction of accumulated As is converted into less reactive thiol complexes or sequestered within vacuoles, the free, toxic As fraction interacting with cytoplasmic enzymes is effectively minimized. Consistent with this possibility, the pronounced accumulation of total As in roots coincided with marked increases in γ-EC, GSH, and PC2–PC4 with S supplementation. These concurrent changes are consistent with the possibility that an enhanced thiol pool may contribute to As detoxification through thiol-associated binding and intracellular retention, as reported for PC-dependent sequestration pathways [15,17,37]. However, because PC synthesis is itself induced by As, the increase in PCs may partly reflect higher As loading rather than a cause of tolerance. Moreover, As speciation, intact As–thiol complexes, PC synthase activity, and the subcellular distribution of As were not examined in the present study. Therefore, the formation or vacuolar sequestration of specific As–thiol complexes cannot be established from our data. Within these limitations, the concurrent increases in γ-EC, GSH, and PCs suggest that enhanced thiol metabolism may contribute to the detoxification and intracellular buffering of elevated As (Figure 4). This expanded buffering capacity provides a possible explanation for the improved growth and reduced oxidative damage observed in S-supplemented seedlings despite their higher total As accumulation (Figure 1, Figure 2 and Figure 3). Consequently, these findings support our central hypothesis that S-mediated As tolerance in rice operates independently of As exclusion, demonstrating that internal thiol-dependent detoxification and antioxidant buffering rather than restriction of As accumulation are the primary determinants of physiological resilience under As stress.

### 4.2. Sulfur Supplementation Alleviates Arsenic-Induced Oxidative Stress

While the elevated ROS and MDA levels at 0 mM S likely reflected the combined effects of S deficiency and As exposure, increasing S supply from 1 to 4 mM significantly reduced these oxidative stress markers relative to the adequate-S condition (1 mM S) under As stress (Figure 3). Multivariate analyses, including heatmaps, clustering, and PCA (Figure 6, Figure 7 and Figure 8) further revealed that As accumulation in roots and shoots was positively correlated with ROS production and negatively correlated with plant height and shoot DW. This pattern aligns with previous reports that As-induced ROS generation impairs plant growth and development [17,38,39,40]. Notably, despite lower As concentrations in leaves than in roots, ROS and MDA levels were markedly higher in leaves (Figure 3), as also reflected in the normalized heatmap (Figure 7). This tissue-specific pattern likely reflects the heightened vulnerability of leaves as the primary sites of photosynthesis and electron transport, where even low As concentrations can disrupt electron flow, generate excess ROS, and reduce chlorophyll content [41]. The relatively lower thiol-rich compound levels in leaves than in roots (Figure 4). In addition, the stronger depletion of Zn and Mn in shoots (Figure 5I,K), which are micronutrients essential for ROS-scavenging enzyme activity, may further amplify the oxidative burden in leaf tissues. Importantly, S supplementation significantly lowered O_2_^•–^, H_2_O_2_, and MDA levels in both roots and leaves compared with As-only treatments (Figure 3). This reduction in oxidative damage was accompanied by sustained growth performance without visible growth inhibition in S-supplemented seedlings. These findings demonstrate that S effectively alleviates As-induced oxidative stress, consistent with previous studies showing that S supplementation reduces ROS accumulation and lipid peroxidation induced by toxic metals in rice and other crops [42,43]. Notably, this reduction in oxidative stress markers was observed despite elevated As accumulation in S-supplemented seedlings, indicating that S confers oxidative stress tolerance through mechanisms other than As exclusion.

### 4.3. Sulfur-Driven Enhancement of the γ-EC–GSH–PCs Cascade Mediates Arsenic Detoxification

Increasing S supply from 1 to 4 mM further increased γ-EC concentrations in both roots and leaves, with a more pronounced response under As stress than under As-free conditions (Figure 4A). γ-EC is the immediate precursor of GSH and is produced through the first and rate-limiting step of GSH biosynthesis [44]. Therefore, its accumulation may reflect increased precursor availability for GSH production and subsequent PC biosynthesis. In addition to serving as a metabolic precursor, γ-EC contains a reactive thiol group that may contribute to cellular redox buffering and the maintenance of the non-protein thiol pool under oxidative stress [44,45,46]. The greater increase in γ-EC in roots than in leaves suggests that thiol metabolism was more responsive in roots under As exposure. Such root-localized stimulation would provide an early biochemical defense by increasing the availability of reduced-S metabolites required for antioxidant protection and As complexation. Notably, this response occurred despite the greater As accumulation observed in S-supplemented seedlings (Figure 2). Thus, enhanced γ-EC production cannot be attributed to reduced As influx but instead reflects an active metabolic adjustment that reinforces thiol-dependent defense under increased internal As loading.

Consistent with the γ-EC response, S supplementation markedly increased GSH concentrations in both roots and leaves, with the strongest increase occurring in roots (Figure 4B). This tissue-specific response indicates that additional S supply enhanced thiol accumulation in roots, the primary site of As uptake [47,48]. Nevertheless, GSH concentrations under As stress remained lower than those under non-stress conditions, even though the γ-EC pool was substantially increased by S supply. The lower GSH concentration under As stress, despite the expanded γ-EC pool, may reflect enhanced GSH utilization for non-enzymatic ROS detoxification and PC biosynthesis, rather than indicating an impaired biosynthetic capacity. GSH is consumed both as a reducing substrate in antioxidant metabolism and as the immediate precursor for PC synthesis. Consequently, As stress may shift GSH metabolism from the maintenance of the free GSH pool toward its rapid utilization for ROS detoxification and As chelation [49,50]. The simultaneous increase in γ-EC and PCs, together with the comparatively smaller accumulation of free GSH, suggests that newly synthesized GSH was rapidly channeled into downstream detoxification processes. This metabolic pattern indicates active engagement of the thiol-based defense system rather than its exhaustion under elevated As accumulation.

The pronounced accumulation of PC2, PC3, and PC4 following S supplementation further supports this interpretation (Figure 4C–E). PCs are synthesized enzymatically from GSH by PC synthase following exposure to As and other toxic elements. Their cysteine-derived thiol groups have been reported to form stable complexes with trivalent As species, facilitating intracellular detoxification and subsequent compartmentation, principally within vacuoles [16]. However, because only total As was measured, the As species present in the tissues of our plants are unknown. The greater accumulation of PCs in roots than in leaves is therefore consistent with the possibility that thiol-dependent complexation contributes to As retention in the tissue where As initially enters the plant. In line with this, Das et al. [50] proposed that enhanced PC production and subsequent intracellular compartmentation of metal-thiol complexes may restrict the mobility of free As species, thereby limiting their translocation to photosynthetically active shoot tissues. In particular, PC2 showed the strongest accumulation in roots, with levels approximately 13.4-fold and 5.1-fold higher than those of PC3 and PC4, respectively, indicating that PC2 was the predominant phytochelatin species accumulated under As with S supplementation. Together with the increases in PC3 and PC4, this pattern suggests that enhanced phytochelatin accumulation may contribute to thiol-mediated As detoxification under S supplementation.

### 4.4. Sulfur-Mediated Nutrient Reallocation Is Associated with Thiol-Dependent Arsenic Detoxification

Beyond the direct stimulation of thiol metabolism, S supplementation markedly altered the tissue-specific mineral nutrient balance of As-stressed rice seedlings, providing a broader physiological context for the observed tolerance response (Figure 5). S supply increased N and K accumulation, particularly in shoots (Figure 5A,C), which may have contributed to the maintenance of metabolic activity and growth under As stress. Notably, S accumulation was strongly enhanced in roots of As-stressed seedlings supplied with S (Figure 5F). This response was consistent with the concurrent increases in γ-EC, GSH, and PCs, suggesting that enhanced S accumulation in roots may have supported thiol metabolism under As exposure, where roots are a major site of As uptake and subsequent physiological responses [51]. A comparable pattern has been reported in As-stressed tomato, where alleviation of As toxicity was associated with coordinated changes of Fe and As absorption, antioxidant capacity, N metabolism, and S assimilation [11].

In contrast, S supplementation decreased the concentrations of several micronutrients in a tissue-dependent manner, particularly Zn and Mn in shoots and Fe and Cu in roots (Figure 5G,I–K). These elements serve as cofactors for antioxidant enzymes, including different SOD isoforms, catalase, and peroxidases [52,53]. Despite these mineral changes, S-supplemented seedlings exhibited lower O_2_^•–^, H_2_O_2_, and MDA levels than seedlings exposed to As alone (Figure 3). Together with the increases in γ-EC, GSH, and PCs, these responses suggest that enhanced thiol metabolism may have contribute to redox regulation and the alleviation of oxidative damage. Ca and Mo concentrations decreased in both root and shoots (Figure 5D,M). In contrast, Mg and Si concentrations remained relatively stable (Figure 5E,H), indicating tissue- and element-specific changes in nutrient distribution under S supplementation. These results indicate that S supplementation induced tissue-specific nutrient reallocation while preferentially increasing root S availability to sustain thiol-dependent As detoxification.

It should be noted that the 15-day hydroponic culture used in the present study captures intrinsic physiological responses in rice seedlings but does not reproduce the complexity of flooded paddy soils. In field environments, soil redox conditions and microbial sulfate reduction can promote As–sulfide precipitation, while root Fe plaque formation and adsorption–desorption processes can substantially alter rhizosphere As bioavailability and speciation [5,19,22]. Moreover, the effects of long-term S application and soil–root microbial interactions over the crop life cycle were not assessed. Therefore, these findings should be interpreted within the context of controlled hydroponic conditions. Field validation is needed to determine how S fertilization interacts with soil biogeochemical processes and affects grain As accumulation.

## 5. Conclusions

**Figure 10:**
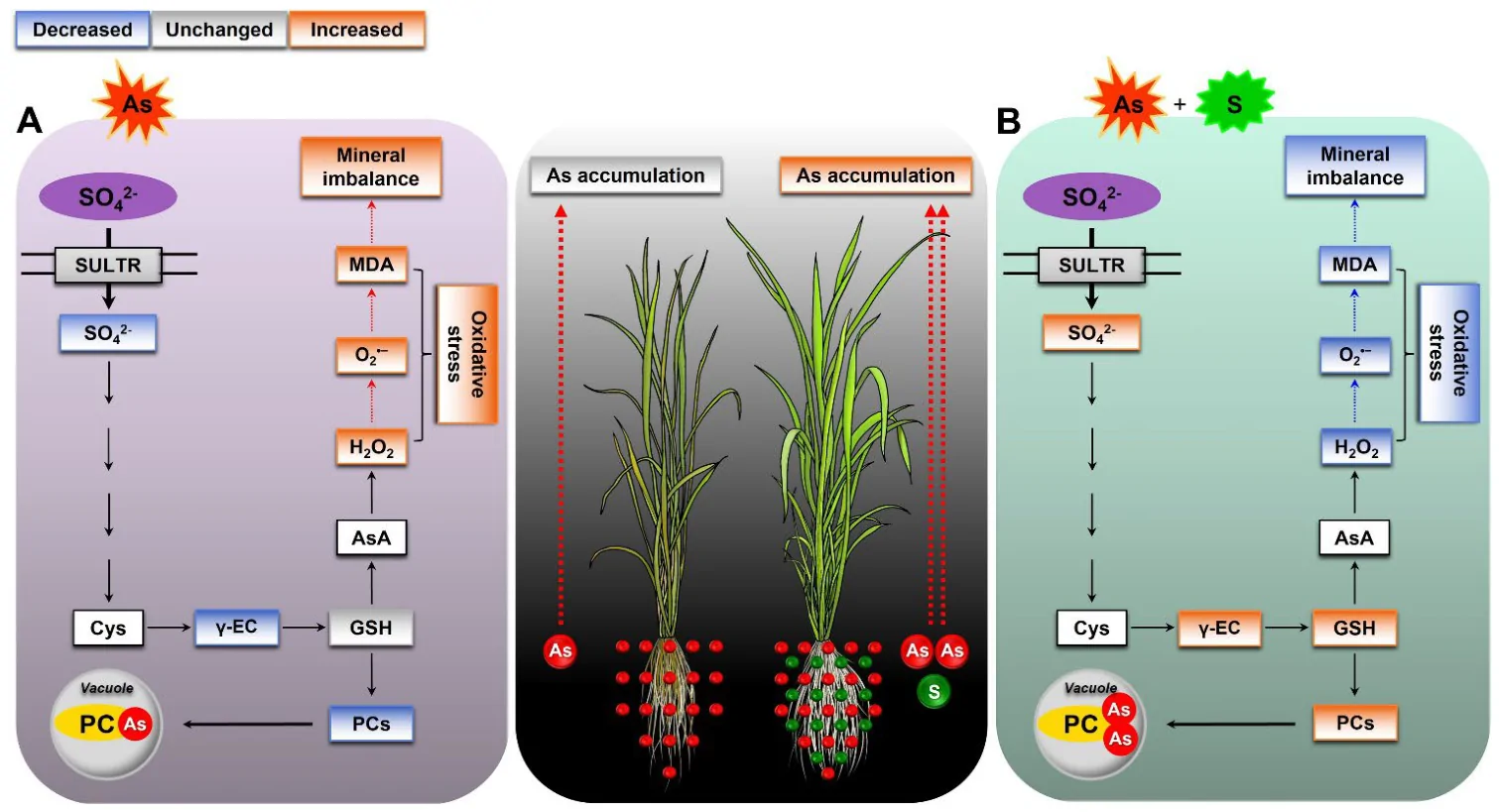
Conceptual diagram summarizing sulfur (S)-mediated physiological, metabolic, and redox responses in rice seedlings under arsenic (As) stress. (**A**) Seedling responses under As exposure alone. (**B**) Seedling responses under As exposure with S supplementation. Experimentally measured parameters quantified in this study are denoted by color-coded status boxes: orange (increased), blue (decreased), and white (unchanged).

This study demonstrates that S supplementation enhances As tolerance in rice seedlings not by restricting total As accumulation, but by increasing the capacity to withstand a greater internal As stress. The coordinated induction of γ-EC, GSH, and PCs, together with reduced oxidative damage (O_2_^•–^, H_2_O_2_, and MDA), suggest that thiol-dependent redox regulation and As detoxification may contribute a central component of this response. S supply also altered nutrient allocation in a tissue- and element-specific manner, suggesting that As tolerance involves an integrated adjustment of thiol metabolism and localized nutrient distribution rather than a uniform improvement in nutrient acquisition. These findings provide evidence that adequate S nutrition can strengthen the physiological resilience of rice to As stress even under conditions of elevated As accumulation. Based on these physiological and biochemical responses, we propose a conceptual model in which S supplementation enhances S assimilation and thiol metabolism, including γ-EC, GSH, and PC production, which may contribute to As detoxification and the alleviation of oxidative damage, thereby supporting plant growth under As stress (Figure 10). However, only total As was quantified in plants nominally supplied with As(III). The in planta chemical forms of As and potential oxidation or reduction dynamics therefore remain unresolved, and the thiol-based interpretations would require speciation analyses to be confirmed. From an agronomic standpoint, the substantial increase in shoot As concentration observed under supplemental S represents a critical caveat regarding agricultural food safety. Because vegetative shoots serve as the primary vascular conduit for developing reproductive panicles, elevated metalloid accumulation in shoot tissues cautions against the indiscriminate application of S fertilizers in As-contaminated paddy fields intended for food production. While flood-tolerant rice biomass might conceptually offer potential in non-food phytomanagement systems where harvested tissues are strictly excluded from the food chain, our findings derive from 15-day hydroponic seedlings and cannot be directly translated into field-scale soil remediation. Therefore, comprehensive full-life-cycle field investigations are essential to determine whether S-induced thiol accumulation arrests As within vegetative nodes or inadvertently facilitates metalloid loading into mature rice grains, thereby ensuring both crop productivity and food safety.

## Data Availability

Data are contained within this article.

## Supplementary Materials

The following supporting information can be downloaded at https://www.mdpi.com/article/10.3390/antiox15101331/s1, Table S1: Final salt composition of the nutrient solutions before As treatment and calculated final major-ion composition of the nutrient solutions; Table S2: F values and P values from two-way ANOVA for the effects of arsenic (As), sulfur (S), and their interaction (As × S) on growth, total As, oxidative stress markers, thiol metabolites, and mineral nutrient contents in roots and shoots of rice seedlings.

## Abbreviations

The following abbreviations are used in this manuscript:

As	Arsenic
S	Sulfur
ROS	Reactive oxygen species
O_2_^•–^	Superoxide anion radicals
H_2_O_2_	Hydrogen peroxide
MDA	Malondialdehyde
GSH	Glutathione
PCs	Phytochelatins
γ-EC	γ-glutamylcysteine
PCA	Principal component analysis
DW	Dry weight
