# Supplementary material for: Sulfur-Induced Thiol Metabolism Confers Arsenic Stress Tolerance Despite Increased Arsenic Accumulation in Rice Seedlings
Source: Antioxidants (Basel). 2026 Oct 15;15(10):1331. doi: 10.3390/antiox15101331 (PMC13701989; doi:10.3390/antiox15101331)
Supplement: Supplementary file 1 [file antioxidants-15-01331-s001.zip › Suppementary Table S2.pdf]

**Supplementary Table S2.** F values and P values from two-way ANOVA for the effects of arsenic (As), sulfur (S), and their interaction (As × S) on growth, total As, oxidative stress markers, thiol metabolites, and mineral nutrient contents in roots and shoots of rice seedlings.

| Variable | Root | | | | | | Shoot | | | | | |
|---|---|---|---|---|---|---|---|---|---|---|---|---|
| | As | | S | | As × S | | As | | S | | As × S | |
| | F value | P value | F value | P value | F value | P value | F value | P value | F value | P value | F value | P value |
| DW | 17.79 | 0.001 | 47.15 | <0.001 | 27.75 | <0.001 | 136.44 | <0.001 | 71.02 | <0.001 | 1.54 | 0.253 |
| Total As concentration | 3235.45 | <0.001 | 152.57 | <0.001 | 152.55 | <0.001 | 2966.87 | <0.001 | 49.91 | <0.001 | 50.17 | <0.001 |
| Total As accumulation[‡] | 2611.07 | <0.001 | 253.96 | <0.001 | 253.93 | <0.001 | 2710.13 | <0.001 | 172.50 | <0.001 | 172.74 | <0.001 |
| $O_2^{\bullet -}$ | 13.62 | 0.003 | 10.14 | 0.003 | 4.00 | 0.047 | 1066.67 | <0.001 | 40.94 | <0.001 | 33.51 | <0.001 |
| $H_2O_2$ | 208.80 | <0.001 | 46.49 | <0.001 | 41.45 | <0.001 | 89.79 | <0.001 | 16.09 | <0.001 | 6.69 | 0.011 |
| MDA | 81.98 | <0.001 | 4.34 | 0.038 | 4.14 | 0.043 | 189.78 | <0.001 | 23.04 | <0.001 | 6.95 | 0.010 |
| γ-EC | 148.59 | <0.001 | 19.17 | <0.001 | 14.07 | <0.001 | 11.32 | 0.006 | 35.84 | <0.001 | 3.17 | 0.078 |
| GSH | 147.99 | <0.001 | 91.10 | <0.001 | 48.51 | <0.001 | 1.30 | 0.276 | 9.03 | 0.004 | 2.74 | 0.105 |
| PC2 | 609.20 | <0.001 | 186.32 | <0.001 | 140.06 | <0.001 | 220.99 | <0.001 | 56.70 | <0.001 | 56.70 | <0.001 |
| PC3 | 249.39 | <0.001 | 57.27 | <0.001 | 18.29 | <0.001 | 1.85 | 0.199 | 0.68 | 0.524 | 0.07 | 0.932 |
| PC4 | 551.09 | <0.001 | 36.66 | <0.001 | 36.66 | <0.001 | ND | ND | ND | ND | ND | ND |
| N | 70.93 | <0.001 | 17.66 | <0.001 | 9.13 | 0.004 | 1724.79 | <0.001 | 152.84 | <0.001 | 32.02 | <0.001 |
| P | 1042.73 | <0.001 | 29.68 | <0.001 | 227.09 | <0.001 | 175.40 | <0.001 | 340.90 | <0.001 | 25.55 | <0.001 |
| K | 32.33 | <0.001 | 16.13 | <0.001 | 0.33 | 0.727 | 0.01 | 0.921 | 47.16 | <0.001 | 18.27 | <0.001 |
| Ca | 62.87 | <0.001 | 42.27 | <0.001 | 1.96 | 0.183 | 7.52 | 0.018 | 89.80 | <0.001 | 12.94 | 0.001 |
| Mg | 224.61 | <0.001 | 5.64 | 0.019 | 34.27 | <0.001 | 7.85 | 0.016 | 11.25 | 0.002 | 10.55 | 0.002 |
| S | 14.66 | 0.002 | 897.86 | <0.001 | 15.64 | <0.001 | 942.52 | <0.001 | 70.65 | <0.001 | 7.49 | 0.008 |
| Si | 3.73 | 0.077 | 2.57 | 0.118 | 2.88 | 0.095 | 2.94 | 0.112 | 1.44 | 0.274 | 1.88 | 0.195 |
| Fe | 7.35 | 0.019 | 82.68 | <0.001 | 15.97 | <0.001 | 8.43 | 0.013 | 2.67 | 0.110 | 10.32 | 0.002 |
| Na | 7.55 | 0.018 | 4.83 | 0.029 | 1.75 | 0.216 | 87.93 | <0.001 | 8.96 | 0.004 | 3.61 | 0.059 |
| Mn | 71.10 | <0.001 | 300.62 | <0.001 | 81.15 | <0.001 | 557.18 | <0.001 | 223.87 | <0.001 | 69.76 | <0.001 |
| Cu | 389.42 | <0.001 | 18.32 | <0.001 | 19.02 | <0.001 | 531.45 | <0.001 | 5.76 | 0.018 | 36.84 | <0.001 |
| Zn | 92.09 | <0.001 | 12.99 | <0.001 | 1.31 | 0.305 | 666.60 | <0.001 | 71.56 | <0.001 | 42.20 | <0.001 |
| B | 28.78 | <0.001 | 42.28 | <0.001 | 11.89 | 0.001 | 0.97 | 0.344 | 8.64 | 0.005 | 2.91 | 0.093 |
| Mo | 22.97 | <0.001 | 293.02 | <0.001 | 19.92 | <0.001 | 127.00 | <0.001 | 5370.48 | <0.001 | 77.72 | <0.001 |

Data were analyzed separately for each tissue (n = 3) with As treatment (control and 15 µM As) and S treatment (0, 1, and 4 mM) as factors, including the As × S interaction. Degrees of freedom: As = 1, S = 2, As × S = 2. P values below 0.001 are shown as <0.001. –, not measured in that tissue; ND, not detected in any treatment.
