# Supplementary material for: Sulfur-Induced Thiol Metabolism Confers Arsenic Stress Tolerance Despite Increased Arsenic Accumulation in Rice Seedlings
Source: Antioxidants (Basel). 2026 Oct 15;15(10):1331. doi: 10.3390/antiox15101331 (PMC13701989; doi:10.3390/antiox15101331)
Supplement: Supplementary file 1 [file antioxidants-15-01331-s001.zip › Supplementary Table S1.pdf]

Supplementary Table S1. Final salt composition of the nutrient solutions before As treatment and calculated final major-ion composition of the nutrient solutions.

Component (salt)	0 mM S	1 mM S	4 mM S	Unit
NH_4NO_3	1.500	1.500	1.500	mM
KH_2PO_4	0.505	0.505	0.505	mM
K_2HPO_4	0.045	0.045	0.045	mM
KCl	4.000	4.000	—	mM
K_2SO_4	—	—	2.000	mM
$CaCl_2$	3.000	3.000	3.000	mM
$MgCl_2$	2.000	1.000	—	mM
$MgSO_4$	—	1.000	2.000	mM
Fe-EDTA	0.400	0.400	0.400	mM
H_3BO_3	14	14	14	µM
$MnCl_2$	5	5	5	µM
$CuCl_2$	0.7	0.7	0.7	µM
$ZnCl_2$	3	3	3	µM
$(NH_4)_6Mo_7O_{24}$	0.7	0.7	0.7	µM
Final ionic concentrations				
SO_4^{2-}	0	1.000	4.000	mM
Cl^-	14.000	12.000	6.000	mM
K^+	4.595	4.595	4.595	mM
Mg^{2+}	2.000	2.000	2.000	mM
Ca^{2+}	3.000	3.000	3.000	mM
NH_4^+	1.500	1.500	1.500	mM
NO_3^-	1.500	1.500	1.500	mM
$H_2PO_4^-$	0.505	0.505	0.505	mM
HPO_4^{2-}	0.045	0.045	0.045	mM
